# Protected Areas: Mixed Success in Conserving East Africa’s Evergreen Forests

**DOI:** 10.1371/journal.pone.0039337

**Published:** 2012-06-29

**Authors:** Marion Pfeifer, Neil D. Burgess, Ruth D. Swetnam, Philip J. Platts, Simon Willcock, Robert Marchant

**Affiliations:** 1 Environment Department, University of York, York, United Kingdom; 2 Center for Macroecology, Evolution and Climate, Department of Biology, University of Copenhagen, Copenhagen, Denmark; 3 WWF-US Conservation Science, Washington, D.C., United States of America; 4 Department of Zoology, University of Cambridge, Cambridge, United Kingdom; 5 School of Geography, University of Leeds, Leeds, United Kingdom; Australian Wildlife Conservancy, Australia

## Abstract

In East Africa, human population growth and demands for natural resources cause forest loss contributing to increased carbon emissions and reduced biodiversity. Protected Areas (PAs) are intended to conserve habitats and species. Variability in PA effectiveness and ‘leakage’ (here defined as displacement of deforestation) may lead to different trends in forest loss within, and adjacent to, existing PAs. Here, we quantify spatial variation in trends of evergreen forest coverage in East Africa between 2001 and 2009, and test for correlations with forest accessibility and environmental drivers. We investigate PA effectiveness at local, landscape and national scales, comparing rates of deforestation within park boundaries with those detected in park buffer zones and in unprotected land more generally. Background forest loss (BFL) was estimated at −9.3% (*17,167* km^2^), but varied between countries (range: −0.9% to −85.7%; note: no BFL in South Sudan). We document high variability in PA effectiveness within and between PA categories. The most successful PAs were National Parks, although only 26 out of 48 parks increased or maintained their forest area (i.e. *Effective* parks). Forest Reserves (*Ineffective* parks, i.e. parks that lose forest from within boundaries: 204 out of 337), Nature Reserves (six out of 12) and Game Parks (24 out of 26) were more likely to lose forest cover. Forest loss in buffer zones around PAs exceeded background forest loss, in some areas indicating leakage driven by *Effective* National Parks. Human pressure, forest accessibility, protection status, distance to fires and long-term annual rainfall were highly significant drivers of forest loss in East Africa. Some of these factors can be addressed by adjusting park management. However, addressing close links between livelihoods, natural capital and poverty remains a fundamental challenge in East Africa’s forest conservation efforts.

## Introduction

Tropical evergreen forests represent around 6% of the terrestrial surface in Eastern Africa, being found mainly in Eastern Congo, Rwanda and Burundi and Eastern Tanzania. They provide goods and services (i.e. natural capital) to rural and urban communities [1], [2], [3], are rich in species and local endemics [4], [5] and are vital carbon sinks, storing from 70 to more than 300 tonnes of carbon per ha, depending on structure, climate and location [6], [7].

East Africa’s evergreen forests also exhibit marked congruence with the most densely populated areas of Africa [8], [9], and may therefore be susceptible to habitat conversion. High human population growth [2], [10] coincides with the expansion of cropland, grazing land and forest plantations at the expense of natural forests [9]. Remaining forests are known to be degraded and declining, particularly in easily accessible coastal areas [11], [12], near main cities [13] and at low altitudes [7]. Towards the eastern edge of the Congo forests and further towards the coast, forests within and outside PAs are increasingly accessible through a network of roads. Additional pressure on forest resources is exerted by commercial timber trade supplying both urban expansion and growing demand from abroad [14]; part of this logging is illegal and thus unregulated [13], [15].

Global and regional analyses suggest that protected areas may be able to stop land clearing and to mitigate logging, hunting, fire and grazing [16]–[19]. Thereby, mixed-used PAs may be as effective, or more effective, than strict PAs in preventing forest fires and forest loss, especially in less remote areas [18], but see Burgess et al [20]. In East Africa, National Parks have firm restrictions on resource use and strong law enforcement [21], although there are exceptions (e.g. Mago National Park in Ethiopia; [22]). Nature Reserves are intended to protect biodiversity, but law enforcement is sporadic and they are often understaffed [21]. Game Parks are largely designed for conservation of large mammals [23] and sport hunting, and are only occasionally patrolled (predominantly during the hunting season). Forest Reserves (commonly gazetted as multi-resource use areas) are often located in areas with valuable timber stocks and used for extractive forestry. They may allow extractive resource use by adjacent communities; this extraction may be permit-regulated, but law enforcement is typically weak [21].

**Figure 1:**
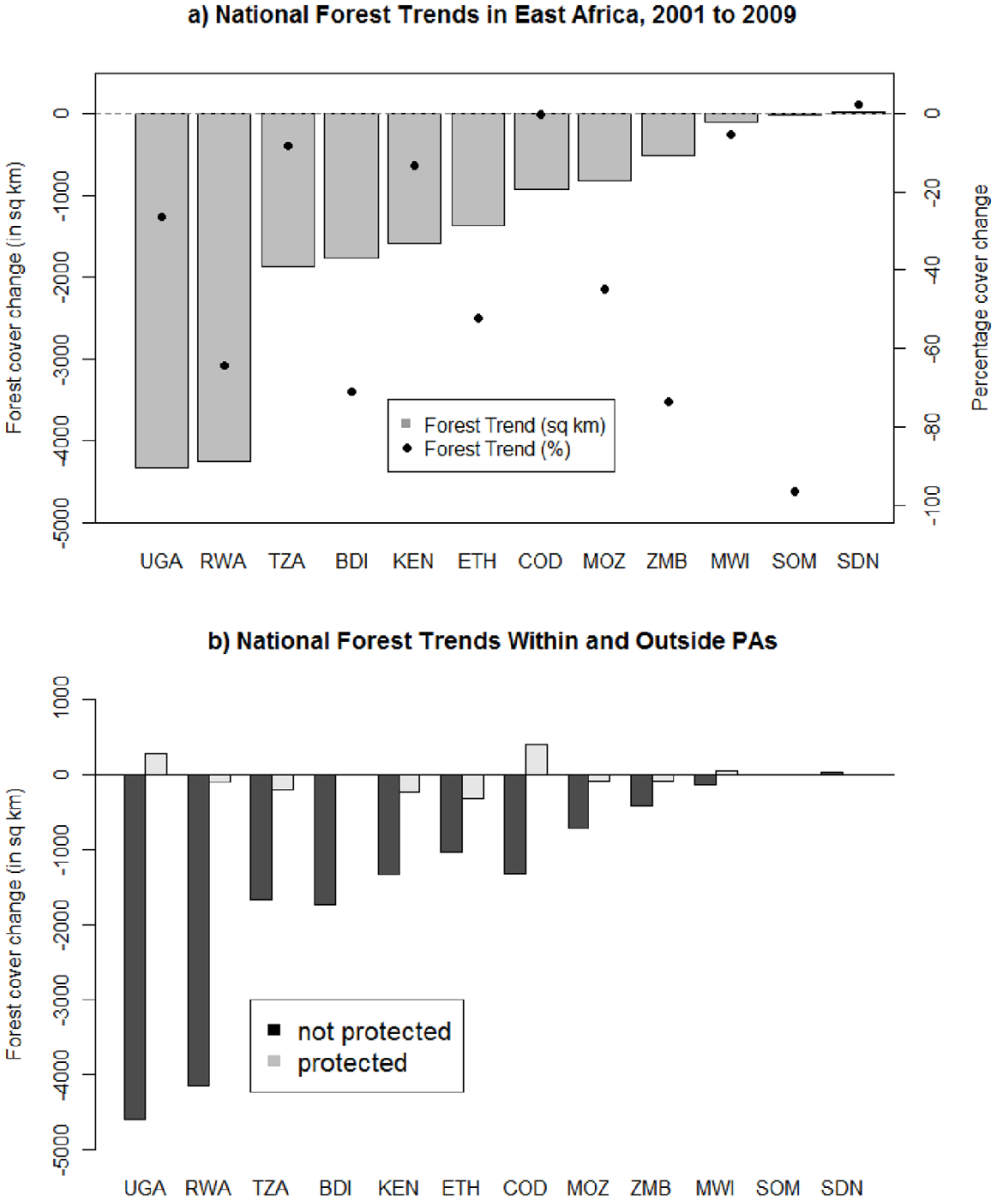
National forest trends in East Africa. Shown are overall forest trends independent of protection status (a) and forest trends depending on protection status (b). Note that only Kenya, Tanzania, Uganda, Rwanda and Burundi are fully covered by the study area.

Analyses of PA effectiveness stress the importance of park management [18], [20] and accessibility [24], [25]. Processes outside PAs (i.e. encroachment of invasive species, regional and local pollution, and socio-economic pressures) may shape processes within PAs [26], [27]. A widespread lack of integration of PAs with local development and community needs can lead to land alienation [28]. Fear of conservation-related ‘land grabs’ may accelerate ‘defensive farming’, as local communities struggling to meet their resource needs expand the land under cultivation to formalize land tenure and gain land use security [29]. Also, ‘leakage’ may offset forest protection within parks by elevating forest loss in areas nearby, as demands for food and fuel still need to be met [30], [31].

**Table 1:** Number of parks per category and country.

	Number of Parks (all parks and parks with forests)		Number of parks with forests and IUCN					
Country	All	Forests	1b	II	III	IV	V	VI
**National Parks**								
Burundi	3	3	0	0	0	3	0	0
Congo^PC^	3	3	0	3	0	0	0	0
Ethiopia^PC^	1	1	0	1	0	0	0	0
Kenya	17	10	0	10	0	0	0	0
Mozambique^PC^	1	1	0	0	0	0	0	0
Malawi^PC^	3	2	0	2	0	0	0	0
Rwanda	3	3	0	2	0	1	0	0
Somalia^PC^	1	1	0	0	0	0	0	0
Tanzania	17	13	0	8	0	2	0	0
Uganda	7	7	0	7	0	0	0	0
Zambia^PC^	10	4	0	4	0	0	0	0
**Nature Reserves**								
Burundi	4	3	0	0	0	3	0	0
Congo	3	3	0	0	0	0	0	0
Tanzania	6	6	1	0	0	0	0	0
**Game Parks**								
Ethiopia	3	1	0	0	0	0	0	1
Kenya	1	0	0	0	0	0	0	0
Mozambique	7	3	0	0	0	1	0	0
Tanzania	29	14	0	0	0	8	0	1
Uganda	5	2	0	0	0	0	0	2
Zambia	15	7	0	0	0	0	0	7
**Forest Reserves**								
Kenya	129	72	0	4	0	0	0	0
Mozambique	5	2	0	0	0	1	1	0
Malawi	26	18	0	0	0	0	0	0
Rwanda	2	2	0	0	0	2	0	0
Tanzania	515	220	6	0	0	45	0	7
Zambia	232	24	0	0	0	0	0	0

PC country only partially covered in the East African study area.

Countries differ strongly with regard to presence and abundance of parks in the different protection categories. For example, Nature Reserves (NR) are only present in three countries.

**Figure 2:**
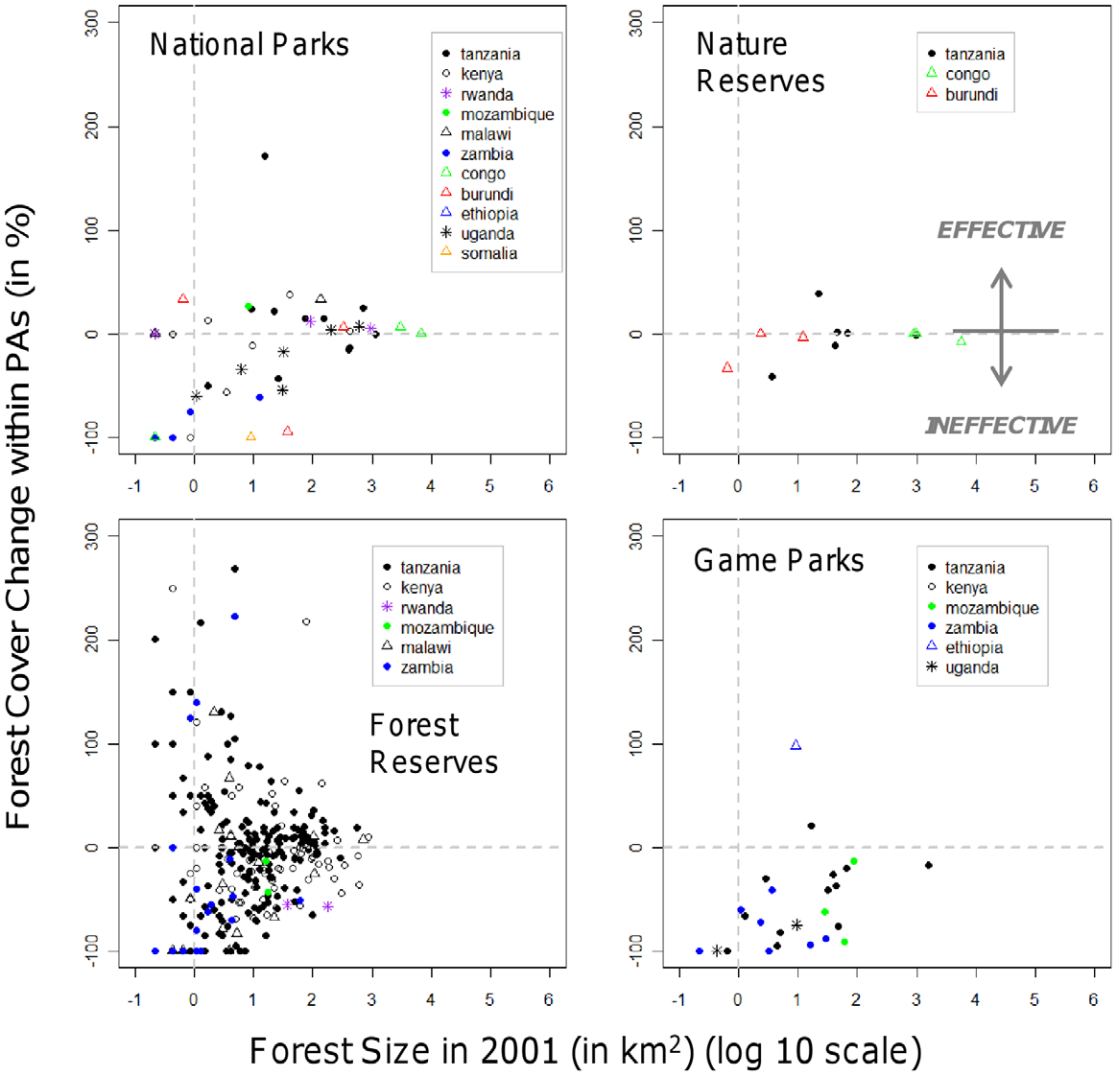
Forest trends within individual parks of four different protection categories between 2001 and 2009 as function of initial forest size in 2001 (log10-scale). For graphical display of forest trends we excluded (very small) PAs that increased their forests by more than 300%. Thus, we excluded five Forest Reserves: Mukugodo FR in Kenya (3.9 km^2^, 822%; forest cover in 2001 and forest change), Ngaia FR in Kenya (0.2 km^2^, 900%), Geita FR in Tanzania (0.2 km^2^, 600%), Vumari FR in Tanzania (0.6 km^2^, 433%), Mwalugulu FR in Tanzania (0.4 km^2^, 450%). On this basis, we also excluded four National Parks: Rubondo NP in Tanzania (0.4 km^2^, 800%), Murchison Falls NP in Uganda (14.5 km^2^, 391%), Mago NP in Ethiopia (0.4 km^2^, 3550%) and Ruma NP in Kenya (1.1 km^2^, 440%).

In our analyses, we evaluate the success of East Africa’s PA network for the conservation of evergreen forests (classified as broad-leaved evergreen tropical forest; International Geosphere Biosphere Programme classification) between 2001 and 2009. We quantify forest trends within PAs, in three buffer zones (B01∶0–1 km from park edge, B15∶1–5 km from park edge, B510∶5–10 km from park edge) and in the unprotected surrounding matrix. By focusing our buffer trend analyses on distances of up to 10 km from park boundaries, we minimize confounding problems of overlap between neighboring PAs. Unlike global scale analyses, which are typically restricted to IUCN category parks [27], our analyses cover all kinds of state managed reserves in East Africa (Forest Reserves, Game Parks, National Parks and Nature Reserves); however, we do not have accurate spatial data for community based management approaches (e.g. Wildlife Management Areas and Village Land Forest Reserves in Tanzania), so these reserves are excluded from analyses. To interpret our findings for forest management, we test for significant dependencies of forest trends on protection status and indicators of human pressure, i.e. population density, road networks, and distance to major towns and fire events.

Joppa et al. [32] have previously evaluated changes in natural vegetation and forest fragmentation across four moist tropical forests: the Amazon and Congo ‘wilderness forests’ and the high-biodiversity forests at the Atlantic coast and in West Africa. They find large geographic variation, however their analyses are static (using a 1 km spatial resolution dataset from 2002) and ignore effects of the matrix at larger spatial scales. In our study, we quantify forest trends at higher spatial resolution (500 m), to produce an assessment of park effectiveness over eight years, based on temporal evidence of forest change within and outside park boundaries, whilst also comparing forest trends within parks and buffers to regional background forest loss (BFL).

**Table 2:** Trends in forest cover across protection categories between 2001 and 2009.

	National Park	Nature Reserve	Forest Reserve	Game Park
***Ineffective***	**22** (96.4±55.3)	**6** (1113.0±919.6)	**204** (28.6±5.3)	**24** (85.5±64.6)
**Size >50% loss**	14 (7.0±3.2)	−	108 (7.7±2.1)	16 (13.1±4.7)
**Size <50% loss**	7 (288.9±156.8)	6 (1113.0±919.6)	91 (54.9±11.0)	8 (230.4±191.2)
***Effective***	**26** (521.4±276.0)	**6** (334.7±190.3)	**133** (41.4±9.7)	**2** (12.8±3.6)
***Forest gain***	23	5	*116*	*2*
***Unchanged***	3	1	*17*	*0*

Shown are the number of parks experiencing forest loss (*Ineffective* PAs) and the number of parks experiencing no change or an increase in forest cover (*Effective* PAs). Values in brackets show the mean area of forests (± standard error; in km^2^). *Ineffective* PAs were further divided into parks that lost more than or less than half their forest cover between 2001 and 2009.

## Results

### Forest Trends

Between 2001 and 2009, forest cover in East Africa decreased in all countries, except the Southern Sudan region (Figure 1), and most severely in the (previously) forest-rich countries of Uganda and Rwanda. Forest decrease was strongest outside protected areas (background forest loss, hereafter BFL) with a decrease of *17,167* km^2^ (−9.3%) over the eight year period. Quantifying forest trends separately for ‘2001 to 2004’ (Period 1) and ‘2004 to 2009’ (Period 2) shows that areal forest loss slowed during Period 2 in some countries (Kenya, Ethiopia, Somalia, Zambia), while in others forest was gained during Period 2 following loss during Period 1 (Rwanda, Burundi, Congo), a pattern broadly consistent inside and outside protected areas (Table S1).

**Figure 3:**
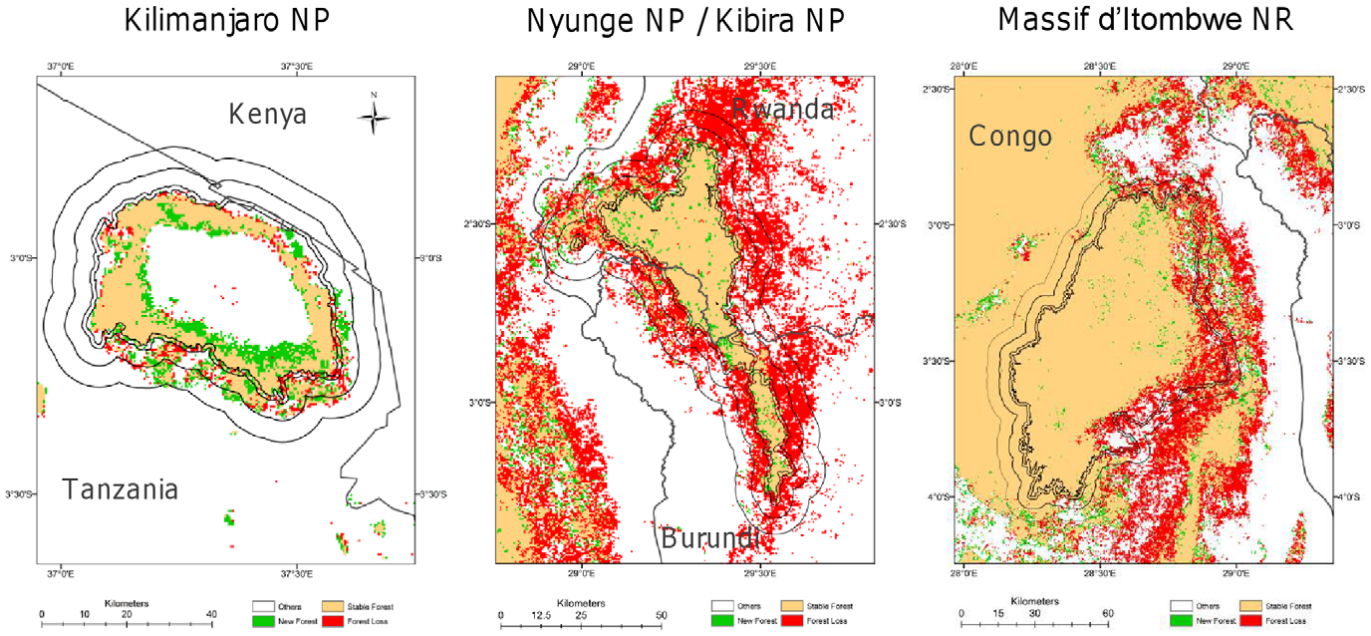
Satellite-derived estimates of forest trend within and around three PAs in East Africa. Forest cover increased (green), decreased (red) or remained constant (orange). Some parks show significant loss in forest cover within their three buffer zones (0–1 km, 1 to 5 km, and 5–10 km). Other land cover transitions are white.

**Table 3:** Percentage of forest change in buffer zones around PAs between 2001 and 2009.

	National Park	Nature Reserve	Forest Reserve	Game Park
***Effective***	100.6±46.4 (20)	8.1±7.6 (5)	90.3±22.0 (64)	44.0±26.9 (3)
B01	62.5±32.9 (18)	−10.8±6.4 (5)	31.4±14.1 (58)	8.8±1.7 (2)
B15	19.4±21.4 (17)	0.0±14.8 (5)	91.8±60.4 (56)	7.4±6.6 (2)
B510	−4.2±17.7 (17)	−15.1±10.3 (5)	19±25.0 (54)	6.4±1.3 (2)
***Ineffective***	−63.4±8.3 (18)	−16.4±6.8 (6)	−57.2±4.0 (83)	−56.4±7.4 (12)
B01	−57.8±15.1 (14)	−29.8±14.1 (6)	−43.6±11.5 (57)	−60.9±10.2 (9)
B15	−11.3±48.2 (14)	−35.8±15.7 (6)	−56.8±5.9 (68)	−52.2±12.6 (10)
B510	−18.1±25.6 (15)	−40.9±17.1 (6)	−40.1±10.7 (62)	−44.7±15.7 (9)

**Buffer zones**: B01: zero to one km from park boundary, B15: one to five km from park boundary, B510: five to 10 km from park boundary. **Cell entries**: Mean values (± standard error) of forest change rates across parks within protection categories are shown (Number of parks in brackets). Note that parks were merged if they were located closer than 10 km from one another.

**Table 4:** Forest trends in buffer zones (B01, B15, and B510) around *Effective* National Parks (i.e. parks that increased or maintained their forest area between 2001 and 2009).

	Buffer zones around Effective National Parks		
Trends in buffers of *Effective*Parks (N – Number of Parks)	B01 (0 to 1 km)	B15 (1 to 5 km)	B510 (5 to 10 km)
N with forest	18	17	17
N (FL)	8	7	10
N (FL > FLBG)	4	3	7
Parks (FL > FLBG)	922 [66.3: −12.2%]	NP6 [2474.6: −12.0%]	NP6 [2329.8: −15.7%]
	NP1 [13.3: −33.9%]	NP5 [952.3: −76.0%]	926 [1.3: −33.3%]
	NP5 [342.7: −31.5%]	9162 [1.1: −100%]	9162 [1.7: −75.0%]
	9162 [3.4: −87.5%]		NP5 [851.3∶82.4%]
			779 [0.4: −100%]
			2296 [0.2: −100%]
			756 [339.9: −23.3%]

**Numbers in bold represent the WDPA Identifier. 756**: Aberdare, Kenya (est. 1950), **779**: Nyika, Malawi (est. 1965), **922**: Kilimanjaro, Tanzania (est. 1973), **926**: Gombe, Tanzania (est. 1968), **2296**: Ruma, Kenya (est. 1983), **9162**: Rusizi, Burundi (est. 1980), **NP1**: merged parks 925 (Arusha, Tanzania, est. 1960) and 303328 (Meru, Tanzania, est. 1951), **NP5**: merged parks 9148 (Nyungwe, Rwanda, est. 1933) and 9161 (Kibira, Burundi, est. 1934), **NP6**: merged parks 863 (Volcans, Rwanda, est. 1929), 18438 (Rwenzori Mountains, Uganda, est. 1991), 40002 (Kibale, Uganda), 40042 (Semuliki, Uganda, est. 1993), 313109 (Mgahinga Gorilla, Uganda, est. 1930), 166889 (Parc National des Virunga, Congo) and 957 (Queen Elizabeth, est. 1952).

N with forest – Number of parks that encompassed evergreen forests in this buffer zone; N (FL) – Number of parks with forest loss in that buffer zone; N (FL > FLBG) – Number of parks with forest loss (FL; in %) that was higher than background forest loss (FLBG; in %) outside protected areas in East Africa; Parks (FL > FLBG) – name of parks with FL > FLBG). See Table 3 for further details on buffer zones.

BFL differed among countries (Table S2), being high in Rwanda (−79.3%; −4,159 km^2^) and Burundi (−82.9%; 1,750 km^2^). Relative BFL was moderate in Uganda (−36.3%), although areal BFL was higher than in any of the other countries (4,609 km^2^). Relative BFL was very low in the Eastern Congo (−0.9%), although that still translated to 1,325 km^2^ of forest lost between 2001 and 2009. Note that relative BFL was also high or moderate in Somalia (85%), Zambia (82%), Ethiopia (56%) and Mozambique (45%). However, our analyses covered only small parts of these countries and areal BFL was low in Ethiopia and Zambia.

**Figure 4:**
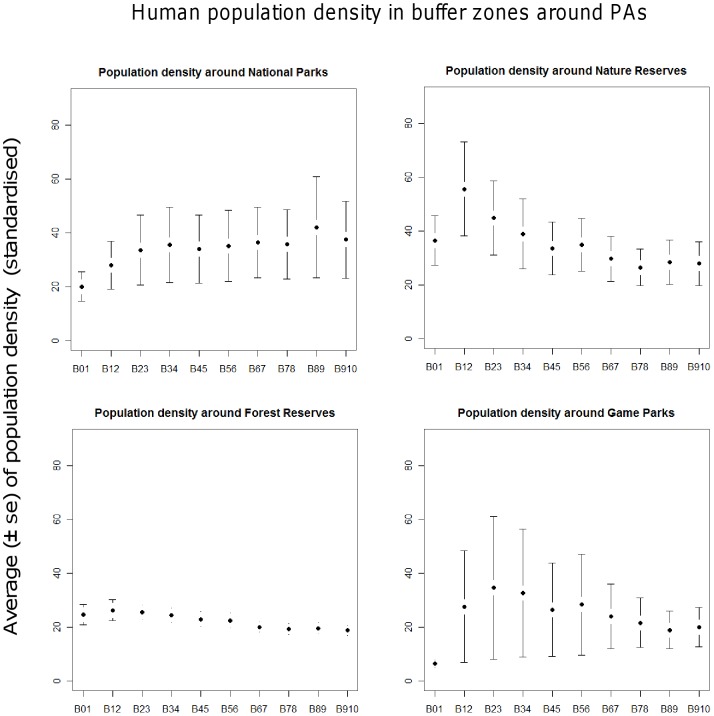
Changes in human population densities with increasing distance from parks across the study area in East Africa. Patterns of human population densities within buffer zones of protected areas differ between protection categories.

**Table 5:** Significant drivers of forest trends (0: no forest loss, 1: forest loss) modelled using general linear models with logit link functions.

	Model 1: East Africa	Model 2: Tanzania, Kenya, Rwanda, Burundi, Congo
**Model**	Population Density (0.021, 0.001)***	Population Density (0.018, 0.001)***
	Distance to Fire (−14.650, 0.649)***	Distance to Fire (−13.940, 0.684)***
	Slope (0.077, 0.009)***	Slope (0.098, 0.009)***
	Game Parks (2.170, 0.252)***	Game Parks (1.704, 0.262)***
	Not Protected (0.840, 0.107)***	Not Protected (0.652, 0.112)***
	National Parks (−0.468, 0.149)**	National Parks (−0.613, 0.159)***
	Nature Reserves (0.041, 0.176)*	Other Protection (−0.376, 0.163)*
	Other Protection (0.219, 0.152)	Distance to Road (−1.302, 0.289)***
	Mean Annual Rain (−0.003, 0.000)***	Distance to Towns (3.878, 0.288)***
		Mean Annual Rain (−0.003, 0.000)***
**LR**	4296.7	3070.8
**Pseudo-R**	0.31	0.22
**AIC**	9586.2	8354
***P***	<0.001	<0.001

Numbers in brackets give the mean and standard error of the coefficient; associated *P* values are given at *P<0.05,

** *P*<0.01,

*** *P*<0.001).

We computed deforestation models for East Africa (Model 1) and for a subset of the study area (Model 2) because geographic data on the spatial location of towns and roads were available for the countries listed in Model 2 only [58]. ‘Protection Status’ is treated as categorical variable with the terms: Game Parks, Not Protected, National Parks, Nature Reserves, Other Protection). A subsequent Wald Chi-Squared test indicates that the overall effect of Protection Status is statistically significant (*P*<0.0001). Abbreviations: likelihood ratio (LR), McFadden’s pseudo R^2^ (Pseudo-R), Akaike Information Criterion (AIC) and significance of model (*P*).

Countries differ in the presence and abundance of reserves within each protection category, as well as in the proportion of IUCN parks (Table 1). Overall, PAs lost comparatively little of their forest cover (378 km^2^; −0.6%), and in Uganda, Congo and Malawi there was an increase in forest cover within protected area boundaries (Figure 1). Game Parks were least effective, losing 24.4% of their 2,078 km^2^ forest cover present in 2001. Nature Reserves and Forest Reserves lost 5.3% and 3.5% of their respective 8,686 km^2^ and 11,337 km^2^ forest area. Only National Parks, often the best protected and funded PAs, increased their forest area, by 3.2% from 15,679 km^2^, although this is primarily driven by the success of National Parks in Tanzania (Figure 2).

PAs differed in their effectiveness depending on protection status, initial forest size and location of the park (Figure 2). The Wilcoxon rank-sum test with Bonferroni adjustment of *P* values (hereafter referred to as *P_bonf_*) indicates significantly higher areal forest loss from Game Parks compared to Forest Reserves and National Parks (*P_bonf_* <0.01), and significantly higher relative forest loss compared to National Parks, Nature Reserves and Forest Reserves (*P_bonf_* <0.01).

Forest conservation success differed considerably within protection categories: nearly 50% of National Parks, 50% of Nature Reserves, 61% of Forest Reserves and 92% of Game Parks were *Ineffective* (Table 2), some of them losing more than 50% of their forests, especially when initial forest extent was small (Table 2). Parks encompassing smaller forest patches experienced stronger relative forest loss (linear model of relative change in forest cover as a function of initial forest size, including only *Ineffective* parks: National Parks: p<0.01; Forest Reserves: p<0.001; Game Parks: p  = 0.066), some losing their forests entirely (61 of 204 *Ineffective* Forest Reserves, 8 of 22 *Ineffective* National Parks, 5 of 24 *Ineffective* Game Parks) (Table 2).

Community benefits programs and resourcing are among the weakest points in protected area management effectiveness [33]. Detailed information on local management of selected PAs in our study area (see Tables S2 and S3) suggests that involving local communities in forest management improves forest conservation outcomes. Mukogodo FR in Kenya, for example, has largely been managed and conserved by the local indigenous community, with little interference of the government following initial monitoring and training [34]. Participatory forest management is also used in Vumari FR in Tanzania, whose legal status is listed as ‘excellent’, and benefits from regular council-funded patrols and conservation interventions such as licensing charcoal burning and pole-cutting. The extent to which PAs can conserve their forests are also likely to be governed by the trade-off between benefits associated with conservation and opportunity costs resulting from forsaken access to forest resources [35]. Economic value (e.g. presence of commercial timber, firewood and charcoal) and local use (e.g. charcoal, honey, grazing, thatching) of forests in Mukogodo FR for example, are low [36].

### Leakage

Ideally, information on forest cover before and after imposing land use restrictions would be used to determine the extent to which gazetting a new PA affects deforestation rates [37]. These analyses are beyond the scope of this study, but we can show that forest loss in the vicinity of some PAs exceeds the 9.3% BFL in East Africa (Figure 3, Table 3). In the B01 buffer, this is the case for 16 National Parks, 7 Nature Reserves, 67 Forest Reserves and 9 Game Parks; in the B15 buffer it is true for 13, 7, 85 and 8 and in the B510 buffer for 16, 7, 78 and 7 National Parks, Nature Reserves, Forest Reserves and Game Parks, respectively. Forest loss in buffer zones of some *Effective* National Parks is higher than BFL in East Africa (Table 4). Country-specific BFL is more severe in most countries than the overall BFL in East Africa (Table S4). There are still some PAs for each protection category and country, where forest loss within buffer zones exceeds country-specific BFL (Table S4).

### Drivers of Forest Loss

Our analyses show that, on average, human density peaks at 1 to 2 km distance from park boundaries for Nature Reserves, Forest Reserves and Game Parks, but increases with distances from the boundaries of National Parks (Figure 4). Nature Reserves, Forest Reserves and Game Parks may be perceived by local communities to represent more viable resources for exploitation (legal or illegally), leading to population clustering in their immediate vicinity, although assessments of perceptions over access to resources need to be carried out within communities to support such conclusion.

As elsewhere [25], [38], human population growth and forest accessibility are significant drivers of the observed forest trends in East Africa. Logit models of the binary response variable ‘no forest loss’/‘forest loss’ show significant correlations with human population density, but also vegetation burning, slope and distance to road networks and towns (Table 5).

Steeper slopes are associated with lower deforestation pressures, presumably because they are less suitable for agriculture and also less accessible compared to gentler slopes [39], [40], which has been linked to lower opportunity costs and lower necessary spend for effective forest protection [40], [41]. Steepness (mean and minimum of slope) was significantly less in the B01 buffer zones of *Ineffective* PAs compared to slope in the B01 buffer of *Effective* PAs, but only in the case of Forest Reserves (Wilcoxon rank sum test: *P*<0.005 and *P*<0.01) and Game Parks (Wilcoxon rank sum test: *P*<0.05 and *P*<0.05). Thus, hampered forest accessibility appears to play a role in reducing deforestation in parks that allow extractive resource use and are generally less well-protected, but is less important in well-protected parks.

We also found a significant effect of long-term rainfall (Table 5), with forests in drier regions appearing more susceptible to habitat conversion. Possible explanations are that dry climates may affect the ability of forests to regenerate after disturbance, that dry forests burn more readily and more extensively [42] and are more suitable for production of charcoal and extraction of firewood. Also, remaining moist forests are often found on challenging mountain terrain.

## Discussion

Large areas of evergreen forests have been lost from East Africa during the 20th century [43], [44], [45] resulting in carbon emissions [6], reduced habitat for forest dependent biodiversity [5], [15], and reduced availability of essential ecosystem services [2], [46]. Initial conservation efforts in East Africa, like elsewhere, focused on creating PAs [20]. However, PAs - worldwide - have faced challenges imposed by inadequate park budgets, varying public and political support and development pressures beyond park boundaries [33], [47].

The mandate for PAs in some East African countries (e.g. Tanzania, Kenya) has changed dramatically in past decades from prioritizing areas for large mammal conservation, to protecting biodiversity in the 1980s and 1990s, and more recently to alleviate poverty and support livelihoods, both key objectives in Tanzania’s Participatory Forest Management (PFM) policy [48]. The proliferation of PFM in Tanzania, legally underpinned by the 1998 National Forest Policy and the 2002 Forest Act, increases decision-making powers of villages on the use of forest resources and empowers them to declare, own and manage their forests [49].

As demonstrated in the case of Mukogodo FR as well as other Effective Nature Reserves and Forest Reserves (S2), PFM can significantly improve forest conservation outcomes. However, human pressure is also shown to lead to deforestation encroachment to within park boundaries, especially among less well-protected parks, and some countries (i.e. Uganda, Burundi, Zambia) fare worse than others. Forest conservation success varies considerably within and between protection categories and within and between countries. And, National Parks perform better than other protection categories in terms of protecting forests.

There is evidence for ‘leakage’ around protected areas (Tables 3, 4, S4), which needs to be analyzed in more detail using higher-spatial resolution satellite images going back to the 1970s, combined with targeted fieldwork for parks established after 1980. Our approach could easily be used to identify ‘sorrow parks’ (i.e. parks that show high deforestation within boundaries and/or within their immediate buffer zones) that should be prioritized for management adjustment. We acknowledge that each of these *Ineffective* parks will require a slightly different, individual management approach. But, comparing management and surrounding matrix traits of these parks to those of *Effective* parks in future analyses can be used to reveal the presence of general mechanisms controlling park effectiveness across geographical scales.

Pragmatic (human-centered) approaches to forest conservation emphasize the importance of conservation in human-modified landscapes [50]. Managing the human-forest interaction across the landscape is politically more feasible than excluding communities from forest resources. However, it may not provide wanted outcomes in regions of rapid human population growth [2], [17], [51], [52] and in regions where the relationship between ‘poor’ and ‘forest use’ is transformed by further interventions (i.e. impact of logging or mining companies, influx of newcomers interested in land for crops and livestock) [53].

While there is an ongoing need to assess ‘fitness-for purpose’ across the PA network, increasing and enforcing existing protection status is likely to remain best-practice on the ground for a while to come. This forest-centered approach to slow or reverse forest loss in East Africa could be combined with (i) establishing and managing multiple-use buffers (e.g. foster tree planting for firewood extraction) around existing PAs [3], [27], (ii) reconnecting local communities to their forests [54], (iii) establishing payments for ecosystem services schemes managed to provide local benefits, and (iv) to identify motivating factors driving resource extraction locally to subsequently provide sustainable and feasible alternatives for services provided by forests [3], [55].

## Materials and Methods

### Study Area

Our study area in Eastern Africa (3,882,887 km^2^; bounded by N6, S-15, W27.5, E42.5), covers Uganda, Kenya, Tanzania, Rwanda and Burundi and extends to partially cover neighboring countries including Somalia, Ethiopia, South Sudan, Congo, Zambia, Malawi and Mozambique.

### Classification of Pas

We defined five land management categories of decreasing protection status: National Parks > Nature Reserves > Forest Reserves > Game Parks > Unprotected land [20]. Boundaries of PAs were derived from the World Database on Protected Areas [56]. Parks were reclassified into National Parks, Nature Reserves, Game Parks (includes Game Reserves, Game Management Areas, Game Controlled Areas, Game Sanctuary, Hunting Reserve and Controlled Hunting Area), and Forest Reserves (includes Village, District and Nationally Managed Forest Reserves). Buffer zones were created around individual PAs after merging parks within protection categories that were located ≤10 km from one another.

### Extraction of Forest Distribution Data

The distribution of evergreen broadleaved forests in the study area was extracted from MODIS Type 1 land cover grids (discussed in Pfeifer et al. [57]; downloaded from https://wist.echo.nasa.gov/~~wist/api/imswelcome/), which provide information on vegetation cover at 500 m spatial resolution. The MODIS algorithm calculates the probability of class membership (PM) for each land cover pixel in each year (via boosting using a base learning algorithm and high spatial resolution Landsat TM imagery). PM is high for evergreen forests (2001 and 2009: PMmedian: 96%, PMmajority: 100%). Background forest loss was calculated as forest cover change between 2001 and 2009 relative to the amount of forest area present in 2001.

### Analysis of Forest Trends

Forest cover trends for the various spatial subsets (PA, PA buffers, national, regional) were computed from maps of evergreen forests between 2001 and 2009. We computed background forest loss for East Africa and separately for each country. We assessed forest trends within individual PAs and their buffers for each protection category, and compared forest trends within park buffers to overall background forest change.

### Environmental Variables in Deforestation Models

Total fire frequency was computed from MODIS active fire hotspot data between 2001 and 2009. We concentrated on fire locations with a reported accuracy ≥50%, accepting that this may result in underestimating fire frequencies. Fire data were converted to 1 km grids, indicating whether a pixel was burned or not in a given year. Fire frequency grids were computed as fire sums between 2001 and 2009 (e.g. fire frequency per pixel could range from 0 to 9). MODIS Burned Area maps (MCD45A1) between 2001 and 2009 were downloaded from http://modis-fire.umd.edu/form.html (discussed in Pfeifer et al. [57]). These grids were transformed into annual presence/absence maps indicating whether a pixel got burned or not. The derived maps were subsequently used to compute grids of pixel-specific annual burning probabilities. Road and town distribution data were derived from the Africover project [58]. Spatial analyses were carried out using ArcGIS v9.3 software. Statistical models and graphics were computed using R v2.11.1 statistical software environment.

### Logit Models of Deforestation

We modelled forest change in East Africa as a function of accessibility (distance to roads, distance to towns, protection status and slope), vegetation burning (annual burning probabilities and pixel-specific fire frequency at annual resolution), and human population density (persons per 500 m cell) derived from density maps at 1 ha spatial resolution [59]. The binary response variable in the logit models of forest change was derived by selecting 5000 points randomly from pixels with forest loss and 5000 points from pixels maintaining their forests. The points were placed more than 1 km distance from one another to minimize spatial autocorrelation [60], [61].

## Supporting Information

Table S1 Comparison of forest loss (in km^2^ and %) within and outside PAs in two time periods (P1∶2001–2004, P2∶2004–2009).
(DOC)

Table S2 Information on management of eight randomly selected *effective* protected areas in East Africa.
(DOC)

Table S3 Information on nine randomly chosen *Ineffective* protected areas in East Africa.
(DOC)

Table S4 Comparison of forest loss within PA and within their buffer zones to country-specific background forest loss (BFL).
(DOC)
