# Supplementary material for: Protected Areas: Mixed Success in Conserving East Africa’s Evergreen Forests
Source: PLoS One. 2012 Jun 29;7(6):e39337. doi: 10.1371/journal.pone.0039337 (PMC3387152; doi:10.1371/journal.pone.0039337)
Supplement: Table S1 — Comparison of forest loss (in km2 and %) within and outside PAs in two time periods (P1∶2001–2004, P2∶2004–2009). (DOC) [file pone.0039337.s001.doc]

**Table S1.** Comparison of forest loss (in km^2^ and %) within and outside PAs in two time periods (P1: 2001-2004, P2: 2004-2009).

| **Country** | **Not Protected - Forest Change** | | | | **Protected - Forest Change** | | | |
| --- | --- | --- | --- | --- | --- | --- | --- | --- |
|  | **P1 km^2^** | **P2 km^2^** | **P1 %** | **P2 %** | **P1 km^2^** | **P2 km^2^** | **P1 %** | **P2 %** |
| Burundi | -1835.1 | 85.6 | -87.0 | 31.13 | -19.7 | 3.9 | -5.3 | 1.1 |
| Eastern Congo | -3539.2 | 2214.3 | -2.5 | 1.59 | -202.1 | 597.0 | -0.5 | 1.4 |
| Ethiopia | -845.6 | -194.2 | -45.3 | -19.05 | -338.6 | 10.9 | -44.8 | 2.6 |
| Kenya | -1377.5 | 35.3 | -28.0 | 0.99 | -136.7 | -106.5 | -2.0 | -1.6 |
| Northern Mozambique | -52.2 | -675.9 | -3.2 | -43.32 | -11.3 | -81.1 | -5.2 | -39.4 |
| Northern Malawi | 216.7 | -357.4 | 28.7 | -36.77 | 485.6 | -447.9 | 42.6 | -27.5 |
| Rwanda | -4517.4 | 358.1 | -86.1 | 49.28 | -139.9 | 33.4 | -10.0 | 2.7 |
| Southern Sudan | 47.1 | -29.7 | 5.8 | -3.44 | 0.2 | -0.4 | 100.0 | -100.0 |
| Southern Somalia | -2.4 | -0.2 | -78.6 | -33.33 | -7.5 | -1.3 | -85. | -100.0 |
| Tanzania | -794.2 | -875.7 | -7.4 | -8.79 | 344.8 | -555.9 | 3.0 | -4.8 |
| Uganda | -2224.2 | -2384.4 | -17.5 | -22.73 | 199.8 | 80.9 | 5.4 | 2.1 |
| North-Eastern Zambia | -365.3 | -53.7 | -71.3 | -36.48 | -44.7 | -44.7 | -25.0 | -33.3 |
