# Supplementary material for: Protected Areas: Mixed Success in Conserving East Africa’s Evergreen Forests
Source: PLoS One. 2012 Jun 29;7(6):e39337. doi: 10.1371/journal.pone.0039337 (PMC3387152; doi:10.1371/journal.pone.0039337)
Supplement: Table S2 — Information on management of eight randomly selected effective protected areas in East Africa. (DOC) [file pone.0039337.s002.doc]

**Table S2** Information on management of eight randomly selected *effective* protected areas in East Africa.

|  | Cat | Name | Coun | Forest in Park (in km^2^) | | Forest in Buffer | | IUCN Status | Est. | Notes |
| --- | --- | --- | --- | --- | --- | --- | --- | --- | --- | --- |
|  |  |  |  | ‘01 | CH | Pres | Loss |  |  |  |
| Effective Parks | NP | Kiliman-jaro | TZA | 705 | 170 | yes | yes | II | 1973 | High tourism numbers, World Heritage Site, Well resourced, Regular border controls, Surroundings heavily populated, Illegal resource uses, Integrated Regional Conservation Plan to reduce dependence on forests**^1, 2^** |
|  | NP | Kibale | UGA | 612 | 43 | yes | yes | II | 1932 | High tourism numbers, Well resourced, Boundaries well-maintained **^3^**, Densely populated surroundings and communities dependent on subsistence agriculture with heavy deforestation ^3, 4^, Wildlife-community conflicts and poaching, Wild Coffee project for local stakeholders ^3^, Community Resources Management ^5^ |
|  | NP | Nyung-we | RWA | 954 | 51 | yes | yes | IV | 1933 | Some tourists, Boundaries marked, Law enforcement after 1980s ^6^, Densely populated in the surroundings and high rural poverty ^7^ |
|  | NR | Amani | TZA | 22 | 9 | yes | no^*^ | - | 1997 | Some tourists, Well-maintained and adequate law enforcement, Local dry fuel wood collection permitted from designated zone ^8^, Research station, Buffer zone for sustainable resource use and community involvement for management ^8^, NGO driven community projects such as cattle and butterfly farming |
|  | NR | Nilo | TZA | 46 | 1 | yes | yes | 1b | 2007 | Few tourists, Well-maintained and strong adequate law enforcement, Close to Amani, Village forest reserves created to increase forest connectivity, good law enforcement; well-funded for training of staff and office, Community awareness programmes and involvement |
|  | NR | Tayna | COD | 900 | 1 | yes | yes | - | NG | Community managed, Established in the context of armed conflict, Management advocates co-existence of human, forests and wildlife, Tayna Community Conservation Programme, Staff sourced locally, Community development projects ^9^ |
|  | FR | Muko-godo | KEN | 4 | 32 | yes | no* | - | 1937 | Mountain forest island with water catchment function, heavy deforestation in the surrounding landscapes in the past, Fire threat and grazing pressure ^10^, Low values for commercial wood products and local forest use ^11^, Local communities (i.e. Masaii) as effective forest custodians, strong CFM ^12^ |
|  | GA | Borana | ETH | 9 | 9 | yes | no | VI | 1973 | Eco-Tourism, Designated as controlled hunting area, Huge park with very low forest cover, Education support programme, Game proof fencing to reduce human-wildlife conflict, Staff predominantly sourced locally ^13^ |

^*^ no loss measured across all cells in buffer, but spatial variation with some cells losing forest; NG – not given; CFM – Community based forest management; PFM – Participatory forest management. Note that Kibale NP was gazetted in 1932 but not formally established until the 1990s.

1. Meyer, A. (2009) Conservation and Development on and Around the slopes of Mount Kilimanjaro, Tanzania. <http://www.stolaf.edu/depts/cis/wp/meyeramj/documents/Kilimanjaro%20Conservation%20and%20Development.pdf> [Accessed May 2012].

2. Clough, DA. UNEP-WCMC. Kilimanjaro National Park, Tanzania. In: Encyclopedia of Earth. Eds. Cutler, J., Cleveland. <http://www.eoearth.org/article/Kilimanjaro_National_Park,_Tanzania> [Accessed Retrieved April 2012]

3. Lilieholm, RJ, Weatherly, WP. (2010) Kibale Forest Wild Coffee: Challenges to Market-Based Conservation in Africa. Conserv Biol. 24: 924-930.

4. Hartter, J, Ryan, SJ, Southworth, J, Chapman, CA (2011) Landscapes as continuous entities: forest disturbance and recovery in the Albertine Rift landscape. Landscape Ecol 26: 877-890.

5. Mugisha, AR, Jacobson, SK (2004) Threat reduction assessment of conventional and community-based conservation approaches to managing protected areas in Uganda. Environ Cons 31: 233-241.

6. Rutagarama, E, Martin, A (2006) Partnership for protected area conservation in Rwanda. The Geographical Journal 172: 291-305.

7. Masozera, MK, Alavalapati, JRR (2011) Forest Dependency and its Implications for Protected Areas Management: A Case Study from the Nyungwe Forest Reserve, Rwanda. Scand J For Res 19: 85-92.

8. Reyes, T, Luukkanen, O, Quiroz, R (2010) Conservation and Cardamom Cultivation in Nature Reserve Buffer Zones in the East Usambara Mountains, Tanzania. J Sustain Forestry 29: 696-715.

9. Kakule, JV (2008) Sustainable Forest Management: The Experience of the Tayna Nature Reserve in the Democratic Republic of Congo. Nature & Faune 23: 22-26. *<* cmsdata.iucn.org/downloads/nzooh_et_al_2008.pdf> [Accessed May 2012].

10. Webala, WP, Muriuki G, Lala F, Bett, A (2006) The small mammal community of Mukogodo Forest, Kenya. Afr J Ecol 44: 363-370.

11. Wass, P (1995) Kenya’s Indigenous Forests: Status, Management and Conservation. IUCN Forest Conservation Programme. SADAG. Cambridge. ISBN 2-8317-0292-5.

12. Ongugo, RA, Osumba, PA, Tuzo, PM (2011) Traditional Forest Use and Institutional Change: Case Study of Loita Community Forest, Narok South District, Kenya. Conference paper, Hyderabad, India. <http://dlc.dlib.indiana.edu/dlc/bitstream/handle/10535/7091/95.pdf?sequence=1> [Accessed May 2012].

13. BORANA. The Green Report. 2007. BORANA Lodge. Associated with Laikipia Wildlife Forum. <http://www.borana.co.ke/borana-green-report.pdf> [Accessed May 2012].
