# Supplementary material for: Protected Areas: Mixed Success in Conserving East Africa’s Evergreen Forests
Source: PLoS One. 2012 Jun 29;7(6):e39337. doi: 10.1371/journal.pone.0039337 (PMC3387152; doi:10.1371/journal.pone.0039337)
Supplement: Table S3 — Information on nine randomly chosen Ineffective protected areas in East Africa. (DOC) [file pone.0039337.s003.doc]

**Table S3** Information on nine randomly chosen *Ineffective* protected areas in East Africa.

|  |  |  |  |  |  |  |  |  |  |  |
| --- | --- | --- | --- | --- | --- | --- | --- | --- | --- | --- |
| *Ineffective* Parks | NP | Ruvubu | BDI | 37 | -36 | yes | yes | IV | 1980 | Used during civil war in 1990s ^14^, Relocation of families after establishment, Heavily populated surroundings and subsistence agriculture, National legislation and 1986 Land Code biased towards active land use ^15^, Poaching, logging, tree harvesting and shifting cultivation ^16^ |
|  | NP | Queen Elizabeth | UGA | 6 | -2 | yes | yes | II | 1952 | Eco-Tourism, High human population growth causing encroachment, poaching, illegal resource use and overharvesting, Villages within park boundaries, Human-wildlife conflicts, Community Resources Management ^5^ but unclear governmental structures for revenue sharing ^17^ |
|  | NP | Udzungwa Mountains | TZA | 1132 | -3 | yes | yes | II | 1992 | Heavily populated surroundings, Extractive frontier landscape ^18^, Adjacent permanent research station, Border patrol increase for law enforcement and recent prohibition of firewood collection caused tension with local communities, Forests have high economic and socio-cultural value ^19^, Expansion of private sugarcane farming in adjacent lowlands caused land shortage decreasing food security |
|  | NR | Massif d'Itombwe | COD | 5644 | -448 | yes | yes | - | - | Civil war outbreaks short-cutting conservation efforts and leading to uncontrolled poaching (e.g. of apes) and deforestation ^20^, Not properly gazetted, No quota on resource extraction and no local law enforcement, No local awareness programmes |
|  | NR | Kilombero | TZA | 1007 | -18 | yes | no^1^ | - | 2007 | Rapid human immigration into Kilombero Valley in recent decades ^21^, Upgraded from forest reserve status to attract more funding and thus enable better protection (deforestation slowed down afterwards) |
|  | FR | Eastern Mau | KEN | 300 | -133 | yes | yes | - | 1941 | Heavy deforestation at landscape scale within the Mau forest complex, Subsistence agriculture with little effort for soil conservation resulting in quick land degradation and soil erosion, Combination of drought and land shortage, Ill-planned settlements and encroachment and weak law enforcement in the past, New conservation strategy plan in action (status: 2009) ^22^ and evictions started 2009 |
|  | FR | Gishwati | RWA | 178 | -101 | yes | yes | IV | 1933 | Civil instability caused heavy deforestation in the 1990s, Small park receiving little post-war attention from NGOs ^23^, Pressure for conversion of land to cultivation ^24^ |
|  | FR | Transmara | KEN | 349 | -62 | yes | yes | - | 1941 | Complex of forest reserves that includes Eastern Mau: forest block with heavy deforestation on the fringes (independent of park boundaries) |
|  | GA | Bangweulu | ZMB | 29 | -26 | yes | yes | VI | 1971 | Game Management Areas in Zambia, established to act as buffer zones to National Parks, Suffers from ecological and socio-economical degradation, Communities within park boundaries and affected by poverty, Low funding, Weak management planning and unsupportive policy structures ^25^ |

^*^ no loss measured across all cells in buffer, but spatial variation with some cells losing forest; NG – not given; CFM – Community based forest management; PFM – Participatory forest management

14. McNeely, JA (2003) Conserving forest biodiversity in times of violent conflict. Oryx 37: 142-152.

15. Beck, J, Citegetse, G, Ko, J, Siber, S (2010) Burundi Environmental Threats and Opportunities Assessment (ETOA). Report for US AID by USDA Forest Service International Programs. <www.encapafrica.org/.../USAID_Burundi_ETOA_Final_PUBLIC_09> [Accessed May 2012].

16. Remegie, N, Yansheng, G (2008) Anthropogenic Impacts on Protected Area of Burundi. Case Study of Ruvubu National Park. The Journal of American Science 4: 26-33. < http://www.jofamericanscience.org/journals/am-sci/0402/04_0373_remegie_am.pdf> [Accessed May 2012].

17. Nampindo, S, Plumptre, A (2005) A socio-economic assessment of community livelihoods in areas adjacent to corridors linking Queen Elizabeth National Park to other protected areas in Western Uganda. Wildlife Conservation Society, Albertine Rift Programme. A report for Conservation International. *<* www.albertinerift.org/Challenges/HumanLivelihoods.aspx> [Accessed May 2012].

18. DeFries, R, Rovero, F, Wright, P, Ahumada, Andelman, S, et al (2010) From plot to landscape scale: linking tropical biodiversity measurements across spatial scales. Front Ecol Environ 8: 153-160.

19. Harrison, P (2006) Socio-Economic Study of Forest-Adjacent Communities from Nyanganje Forest to Udzungwa Scarp: A Potential Wildlife Corridor. Incorporating Livelihood Assessments and Options for Future Management of Udzungwa Forests. WWF Tanzania. Critical Ecosystem Partnership Fund. *<*cepf.tfcg.org/downloads/SocioEco_Udz_Scarp.pdf > [Accessed April 2012].

20. Chamberlan, C, Courage, A, Dunn, A, Dupain, J, Goldsmith, DML, et al (2005) Conservation of Gorillas and Chimpanzees in Itombwe. Gorilla Journal. Journal of Berggorilla & Regenwald Direkthilfe. <http://www.berggorilla.de/fileadmin/gorilla-journal/gorilla-journal-30-english.pdf> [Accessed May 2012].

21. Jones, T, Epps, C, Mbano, B, Coppolillo, P, Mutayoba, B, Rovero, F (2007) Maintaining Ecological Connectivity between the Protected Areas of south-central Tanzania: Evidence and Challenges. <http://fw.oregonstate.edu/pdfs/Jonesetal_Corridors_TAWIRI2007_FINAL.pdf> [Accessed May 2012].

22. Interim Coordinating Secretariat, Office of the Prime Minister, Government of Kenya (2009) Rehabilitation of the Mau Forest Ecosystem. <http://www.kws.org/export/sites/kws/info/maurestoration/maupublications/Mau_Forest_Complex_Concept_paper.pdf > [Accessed May 2012].

23. Hanson, T, Brooks, TM, da Fonseca, GAB, Hoffmann M, Lamoreux JF, et al ( 2009) Warfare in Biodiversity Hotspots. Conserv Biol: 23: 578-587.

24. Andrew, G, Masozera, M (2010) Payment of Ecosystem Services and Poverty Reduction in Rwanda. Journal of Sustainable Development in Africa 12: 122-139.

25. Simasiku, P, Chapoto, A, Richardson, R, Sichilongo, M, Tembo, G, et al (2008) Natural Resource Management, Food Security, and Rural Development in Zambia: Moving from Research Evidence to Action. Working Paper 44. http://www.aec.msu.edu/fs2/zambia/wp44.pdf [Accessed May 2012].
