# Supplementary material for: Protected Areas: Mixed Success in Conserving East Africa’s Evergreen Forests
Source: PLoS One. 2012 Jun 29;7(6):e39337. doi: 10.1371/journal.pone.0039337 (PMC3387152; doi:10.1371/journal.pone.0039337)
Supplement: Table S4 — Comparison of forest loss within PA and within their buffer zones to country-specific background forest loss (BFL). (DOC) [file pone.0039337.s004.doc]

**Table S4.** Comparison of forest loss within PA and within their buffer zones to country-specific background forest loss (BFL).

| **Country** | **Background Forest Loss; BFL** | | **National Parks; N; N with Forest Loss exceeding BFL** | | | | | **Nature Reserves; N; N with Forest Loss exceeding BFL** | | | | | **Forest Reserves; N; N with Forest Loss exceeding BFL** | | | | | **Game Parks; N; N with Forest Loss exceeding BFL** | | | | |
| --- | --- | --- | --- | --- | --- | --- | --- | --- | --- | --- | --- | --- | --- | --- | --- | --- | --- | --- | --- | --- | --- | --- |
|  | **%** | **Km^2^** | N | In | B01 | B15 | B510 | N | In | B01 | B15 | B510 | N | In | B01 | B15 | B510 | N | In | B01 | B15 | B510 |
| **SOM**^PC^ | -85.71 | -2.6 | **1** | 1 | 1 | 1 | 0 | **-** | - | - | - | - | **-** | - | - | - | - | **-** | - | - | - |  |
| **BDI** | -82.91 | -1749.5 | **3** | 1 | 2 | 2 | 1 | **3** | 0 | 0 | 0 | 0 | **-** | - | - | - | - | **-** | - | - | - |  |
| **ZMB**^PC^ | -81.76 | -419.0 | **4** | 2 | 1 | 2 | 1 | **-** | - | - | - | - | **24** | 12 | 0 | 4 | 4 | **7** | 4 | 1 | 1 | 0 |
| **RWA** | -79.32 | -4159.3 | **3** | 0 | 0 | 0 | 1 | **-** | - | - | - | - | **2** | 0 | 0 | 1 | 0 | **-** | - | - | - | - |
| **ETH**^PC^ | -55.75 | -1039.8 | **1** | 0 | 0 | 0 | 0 | **-** | - | - | - | - | **-** | - | - | - | - | **1** | 0 | 0 | 0 | 0 |
| **MOZ**^PC^ | -45.15 | -728.1 | **1** | 0 | 0 | 0 | 0 | **-** | - | - | - | - | **2** | 0 | 1 | 2 | 1 | **3** | 2 | 2 | 2 | 0 |
| **UGA** | -36.25 | -4608.5 | **7** | 2 | 1 | 1 | 1 | **-** | - | - | - | - | **-** | - | - | - | - | **2** | 2 | 0 | 0 | 0 |
| **KEN** | -27.23 | -1342.2 | **10** | 4 | 2 | 1 | 3 | **-** | - | - | - | - | **71** | 23 | 5 | 7 | 8 | **-** | - | - | - |  |
| **MWI**^PC^ | -18.63 | -140.7 | **2** | 0 | 0 | 0 | 1 | **-** | - | - | - | - | **18** | 10 | 7 | 7 | 6 | **-** | - | 1 | 1 | - |
| **TZA** | -15.53 | -1669.9 | **13** | 4 | 5 | 2 | 3 | **6** | 1 | 2 | 2 | 2 | **220** | 95 | 41 | 54 | 53 | **13** | 12 | 1 | 1 | 3 |
| **COD**^PC^ | -0.93 | -1324.9 | **3** | 1 | 1 | 1 | 1 | **3** | 1 | 1 | 1 | 2 | **-** | - | 3 | 2 | 1 | **-** | - | 1 | 1 |  |
| **SDN**^PC^ | 2.12 | 17.3 | **-** | - | - | - | - | **-** | - | - | - | - | **-** | - | - | - | - | **-** | - | - | - |  |

## ^PC^ country only partially covered in the East African study area.

N – Number of parks in that category. In – Number of parks where forest loss rate within park boundaries exceeded background forest loss rate. B01, B15, B510 – Number of parks where forest loss rate in buffer zones B01, B15 and B510 exceeded country-specific background forest loss rates. BDI – Burundi, COD – Eastern Congo, ETH – Southern Ethiopia, KEN – Kenya, MOZ – Northern Mozambique, MWI – Northern Malawi, RWA – Rwanda, SDN – Southern Sudan, SOM – Southern Somalia, TZA – Tanzania, UGA – Uganda, ZMB – North Eastern Zambia.
